# Cervical cancer prevention in Burkina Faso: a stakeholder’s collaboration for the development of awareness messaging

**DOI:** 10.3389/fonc.2024.1383133

**Published:** 2024-05-10

**Authors:** Samiratou Ouedraogo, Assanatou Bamogo, Georges Tiendrebeogo, Simon Kaboré, Anne-Marie Turcotte-Tremblay, Mamoudou Maiga, Samdapawindé Thérèse Kagoné, Olga Mélanie Lompo, Nicolas Meda

**Affiliations:** ^1^ Oliver Reginald (OR) Tambo Africa Research Chair “Research and Action Against Cancer”, Faculty of Health Sciences, University Joseph KI-ZERBO, Ouagadougou, Burkina Faso; ^2^ Observatoire National de la Santé de la Population (ONSP), Institut National de Santé Publique (INSP), Ouagadougou, Burkina Faso; ^3^ Department of Public Health, Faculty of Health Sciences, University Joseph KI-ZERBO, Ouagadougou, Burkina Faso; ^4^ Department of Global and Public Health, McGill School of Population and Global Health, McGill University, Montreal, QC, Canada; ^5^ Department of Maternal and Child Health, University of North Carolina at Chapel Hill, Chapel Hill, NC, United States; ^6^ Johns Hopkins University, Bloomberg School of Public Health, Baltimore, MD, United States; ^7^ Association d’Anthropologie Médicale et de la Santé (AMADES), Ouagadougou, Burkina Faso; ^8^ Réseaux Accès aux Médicaments Essentiels, Ouagadougou, Burkina Faso; ^9^ Vitam - Centre de Recherche en Santé Durable, Université Laval, Québec, QC, Canada; ^10^ Center for Global Oncology, Northwestern University, Chicago, IL, United States; ^11^ Centre MURAZ, Institut National de Santé Publique, Bobo-Dioulasso, Burkina Faso; ^12^ Laboratory of Pathological Anatomy and Cytology, Yalgado Ouedraogo University Hospital, Ouagadougou, Burkina Faso

**Keywords:** cancer prevention, cervical cancer, cancer awareness, stakeholder engagement, Burkina Faso

## Abstract

**Background:**

Cervical Cancer stands as the second leading cause of both incident female cancers and deaths in Burkina Faso. Unfortunately, the prevention, early detection, and care of cervical cancers are suboptimal at individual, institutional, and national levels. In October 2023, we organized a stakeholder’s workshop to develop cervical cancer awareness messaging for disease control in the country.

**Methods:**

A one-text workshop was organized with stakeholders working toward improving health in general or women’s health and well-being. A participatory, learning, and adaptive approach was used to facilitate discussions and activities, ensuring the contribution of all participants. Contextual evidence-based and empirical elements about cervical cancer burden and preventive strategies were presented to the participants by key informants. These served as the foundation for a collaborative formulation of messaging content that aimed at raising awareness about cervical cancer.

**Results:**

Sixty-two participants from 28 organizations attended the workshop. They work mainly at local and international non-governmental organizations, civil society organizations, universities, university hospitals, research centers, and the Ministry of Health. During the first and second days of the workshop, the participants explored cervical cancer data, its preventive and treatment options available in Burkina Faso, communication strategies for behavioral change, and determinants of the use of prevention and health promotion services. During the following three days, 3 working groups were formed to define strategies, and key messages adapted to diverse tools and targeted audiences. All information was validated during plenary sessions before the end of the workshop and available to all participants and their organizations for cancer awareness activities.

**Conclusion:**

Upon conclusion of the workshop, the participants provided insightful information for the development of cervical awareness messaging in Burkina Faso. They formed the first community of practice to serve as a dynamic platform for implementation, monitoring, evaluation, and continued learning activities.

## 1 Introduction

In 2022, it is estimated that 1,308 cases and 1,018 deaths due to CC occurred in Burkina Faso, positioning this disease as the second most prevalent cancer and an important public health challenge (1). The burden of CC in Burkina mirrors the broader context of Sub-Saharan Africa (SSA) and is influenced by many factors such as i) a higher risk of Human Papilloma Virus (HPV) infection due to poverty, risky sexual behavior, and low socioeconomic status, coupled with inadequate HPV vaccination coverage ii) an absence of effective screening programs; iii) a lack of awareness about CC prevention and early detection benefits, iv) an inequitable access to healthcare services (2–8). Yet, as part of a national policy of free healthcare for children under five and women launched in March 2016, cervical cancer screening and treatment have been decreed free for all women in Burkina Faso (9). Moreover, on April 26, 2022, the Ministry of Health and Public Hygiene introduced the HPV vaccine in the country’s Expanded Program on Immunization (EPI) targeting nine-year-old girls (10, 11). These strategies align with the World Health Organization’s (WHO) global strategy aimed at eliminating cervical cancer within generations. Indeed, the WHO strategy delineates specific objectives, including achieving a 90% HPV vaccination rate among girls by age 15, a 70% screening rate among women at key ages, and a 90% treatment rate for those identified with cervical disease, all by 2030 (12). Many research has already shown the importance of the WHO strategy in contributing to CC elimination (7, 13–16). But to meet these WHO targets, effective dissemination of information and promotion of preventive measures are vital. An impactful communication strategy development for consistent messaging should include collaboration among all stakeholders interested in CC elimination such as researchers, health professionals, advocacy groups, journalists, etc (9, 17–23). It encompasses: i) accessible HPV vaccination and cervical screening information for all population segments; ii) comprehensive and tailored information matching the literacy levels of the target audience; iii) client-centered communication addressing the needs of diverse sub-groups; iv) health professionals’ proficiency in screening and communication.

This paper outlines the planning process and key findings of a stakeholder workshop in Burkina Faso, designed to foster effective awareness messaging for cervical cancer control. The workshop brought together healthcare professionals, government officials, community leaders, patient advocates, non-governmental and civil society organizations, cancer survivors, caregivers, and advocates. It represents a concerted effort to address the unique challenges of CC elimination in Burkina Faso and highlights collaborative approaches that could serve as a model for similar initiatives in other SSA countries.

## 2 Materials and methods

### 2.1 Study type

The work consisted of a participatory approach involving a diverse group of stakeholders who are directly or indirectly interested in/(affected by) cervical cancer. The data collected were qualitative information generated during active engagement and dialogue between participants who attended a five-day workshop.

### 2.2 Workshop participants

From October 2 to 6, 2023, we convened a diverse group of key stakeholders in Burkina Faso to discuss communication strategies for cervical cancer awareness. This group included healthcare professionals, government health officials, community leaders, patient advocates, representatives from non-governmental and civil society organizations specializing in women’s health, as well as cancer survivors, caregivers, and advocates. Stakeholders were encouraged to extend invitations to other interested parties, broadening the workshop’s reach and diversity of perspectives.

The list of stakeholders invited to this workshop was prepared with the support of the Non-Governmental Organization Permanent Secretary in Burkina Faso. It included non-governmental organizations (NGOs) and civil society organizations (CSOs) that were active in communication and sensitization at the community level during the Corona Virus-19 (COVID-19) pandemic as well as those with known interest in cancer, women’s health and well-being, and mental health. Governmental organization representatives were represented mainly by healthcare workers from the main university hospitals of the country (one gynecological medical doctor and two nurses, two oncologists, a pediatrician, two anatomopathologists, and three general practitioners).

We sent special invitations to researchers and clinicians with renowned work experience on cancer in Burkina Faso to present the theoretical concepts of cancer, and the focus of their work and contribute to the workshop discussions. We also invited a cervical cancer survivor identified among our close relatives to bring her experience and voice to the discussion. A detailed list of the organizations invited is presented in **Table 1**.

**Table 1:** List of stakeholders invited to a workshop for the development of awareness messaging for cervical cancer prevention in Burkina Faso.

Category	Organization
Governmental organizations	Ministry of health and Public Hygiene
	National Public health Institute
	Yalgado Ouedraogo University Hospital in Ouagadougou
	Charles De-Gaulle Pediatric University Hospital in Ouagadougou
	Sourô Sanou University Hospital in Bobo
	Bogodogo University Hospital in Ouagadougou
Academic	Joseph KI-ZERBO University in Ouagadougou
	Saint Thomas d’Aquin University in Ouagadougou
Private sectors	Language and design Communication Agency
	Mousso News
	Webactubf.info
Local non-governmental organizations	KIMI Foundation
	Pananetugri Initiative for the Well-being of Women
	Organization for New Initiatives in Development and Health
Civil society organizations	Access to Essential Medicines Network
	Association Noug Yen Ka Woukd Zoom
	African Youth Health and Development Network in Burkina Faso
	Action group against cancer
	Women in Global Health Francophone West Africa
	Brigade rose
	The Support Network for Health Mutuals in Burkina Faso
	Union of Religious and Traditional Leaders of Burkina for Health and Development
International non-governmental organizations	U.S. Agency for International Development
	Union for International Cancer Control
	Médecins du Monde
	Jhpiego
	Marie Stopes International
Cancer patients	Two cancer patients, two survivors
Experts (Moderators)	4 experts in public health and social behavior change

### 2.3 Organization and process

**Figure 1:**
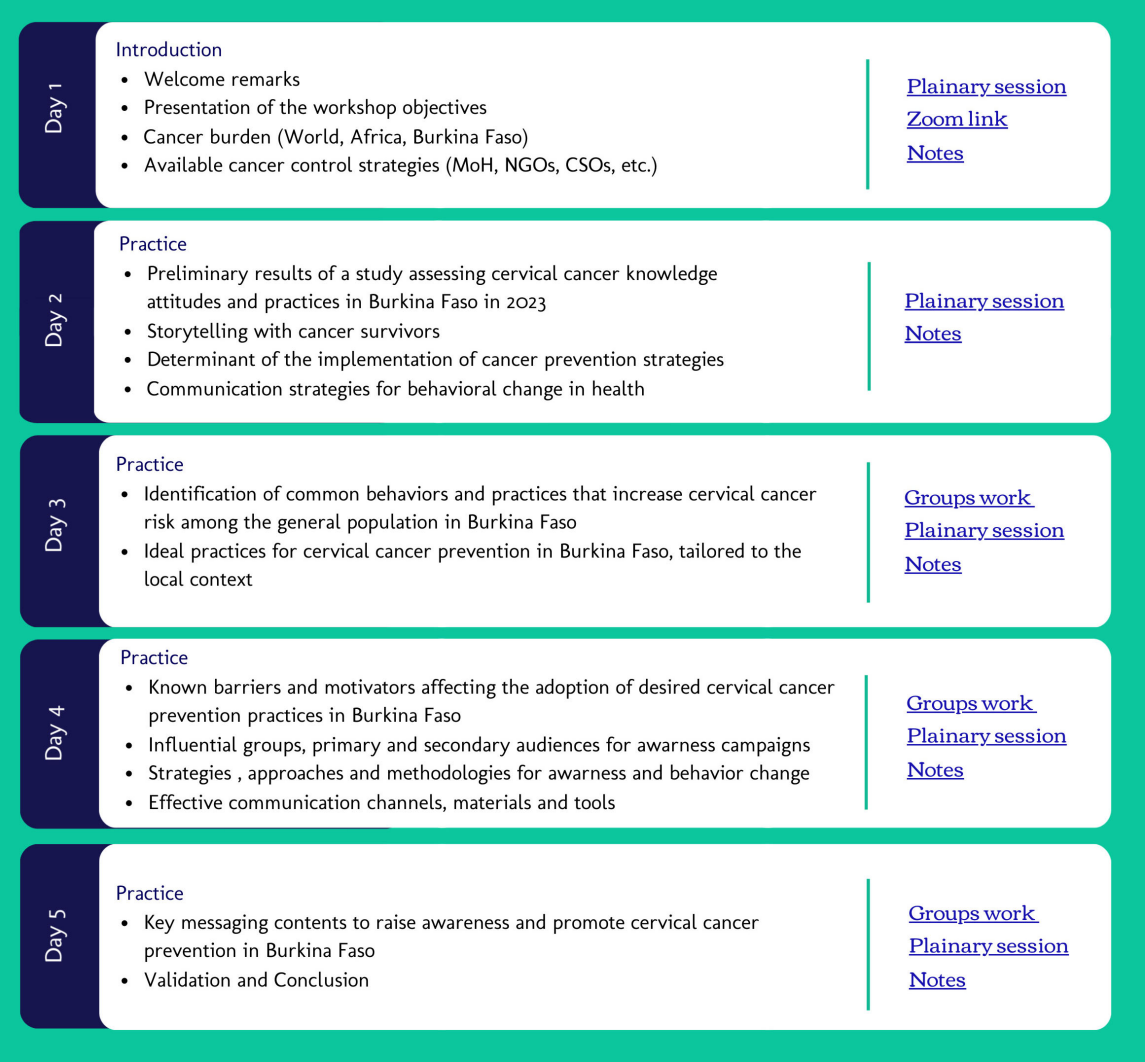
Agenda for a stakeholder’s collaboration workshop for the development of awareness messaging on cervical cancer prevention in Burkina Faso.

Invitations for the workshop were issued on August 12, 2023, to the official email address or contact person of all identified stakeholders. The email included a request to confirm the organization’s interest in attending the workshop through the completion of an online registration form which included the names, roles in the organization, and contact details of their representatives. To maintain engagement and ensure a high turnout, we sent weekly reminder emails leading up to the event. The last reminder included a formal invitation letter, the reference terms of the workshop, and a tentative agenda for the meeting (**Figure 1**).

### 2.4 Workshop setting and logistics

The workshop took place physically at Joseph KI-ZERBO University’s main location in Ouagadougou. We used an online platform on the afternoon of the first day to allow a representative of the International Union for Cancer Control (IUCC) to present their work.

During small group working sessions, we provided a handout (Table 2 template) to guide the discussion and collect the outputs.

### 2.5 Workshop moderators and main speakers

The workshop moderators were four resource persons - two females and two males - identified for their expertise in health and their work with multiple stakeholders:

- The first moderator was a senior public health consultant, trained as a medical doctor with experience in communication for behavior change. He has worked for diverse national and international institutions and organizations in many countries for decades;
- The second moderator was a researcher and lecturer with a master’s and Ph.D. in public health focus on cancer issues and almost 15 years of work experience;
- The third moderator was a researcher and lecturer in sociology and anthropology, with expertise in research ethics and stakeholder collaborations;
- The fourth moderator was a senior consultant with extensive experience in communication for behavior change who provides facilitation services to local and international organizations.

We also invited guest speakers to present general cancer information and discuss their work related to the disease:

- An epidemiologist who presented CC statistics (in the world, in Africa, and Burkina Faso), risk factors, and the strategy for its elimination;
- A gynecologist and specialist in female cancer who provided information on the most frequent female cancers and available treatment options in Burkina Faso;
- A representative of the Ministry of Health and Public Hygiene from Burkina Faso presented the national HPV immunization strategy;
- Representatives from, the Coalition Against Cancer in Burkina Faso, the KIMI Foundation, Médecins du Monde France, and the IUCC presented their organization’s cancer control strategies;
- A researcher who presented the preliminary result of a research on the general population and health professional Knowledge, attitude, and practice about cervical cancer prevention;
- A communication expert who presented on communication strategies for behavioral change;
- A public health expert who presented on the determinants of the use of prevention and health promotion services.

### 2.6 Workshop structuration

The workshop was structured into three main sessions. The initial session provided an overview of the epidemiology of cervical cancer in Burkina Faso, including current control strategies.

The subsequent session was multi-faceted, encompassing:

- Presentations on the barriers and facilitators influencing the adoption of best practices for cervical cancer prevention;
- Discussions on the contextual evidence underlying the development of intervention strategies, content, communication messages, and educational materials;
- A storytelling session where breast, cervical, and ovarian cancer patients and survivors along with their relatives, shared personal experiences related to their journey and battles with the disease.

The latter part of the workshop consisted of four alternating working group sessions, each culminating in a plenary session. The working group sessions focused on the multifaced steps that should be considered for raising awareness for cervical cancer prevention in Burkina Faso and include:

- Common behaviors and practices that increase cervical cancer risk among the general population in Burkina Faso;
- Ideal practices for cervical cancer prevention in Burkina Faso, tailored to the local context;
- Known barriers and motivators affecting the adoption of desired practices;
- Influential groups for primary and secondary audiences for awareness campaigns;
- Strategies, approaches, and methodologies for awareness and behavior change;
- Effective communication channels, materials, and tools;
- Key messaging content to raise awareness and promote cervical cancer prevention.

### 2.7 Data collection and validation

Each working group was led by a moderator and a note-taker. Each group session lasted a minimum of 3 hours and was followed by a plenary session the same or the following day.

The plenary sessions aimed at sharing, discussing, synthesizing, and validating the information presented by the working groups. This strategy aimed at fostering a cohesive and comprehensive understanding among participants and the validation of the content by all.

### 2.8 Ethical considerations

The cancer survivors and their relatives provided their verbal consent to share their stories and experiences and to be audio-recorded for a faithful transcription of the storytelling session. To ensure their confidentiality, no picture was captured.

### 2.9 Feedback mechanisms

At the end of the workshop, all participants were asked to provide verbal and open written feedback regarding the workshop.

### 2.10 Follow-up actions

The workshop ended with all participants agreeing to be part of a virtual Community of Practice for Cancer Control in Burkina Faso.

## 3 Results

### 3.1 Participation in the workshop

Sixty-two participants from 28 organizations (**Table 1**) working toward improving health in general or women’s health and well-being attended the workshop. They were from diverse backgrounds and levels of implication in cancer control and health improvement at local, national, and international levels.

### 3.2 Summary of the discussions of a stakeholder’s workshop for the development of awareness messaging for cervical cancer prevention in Burkina Faso

**Figure 2:**
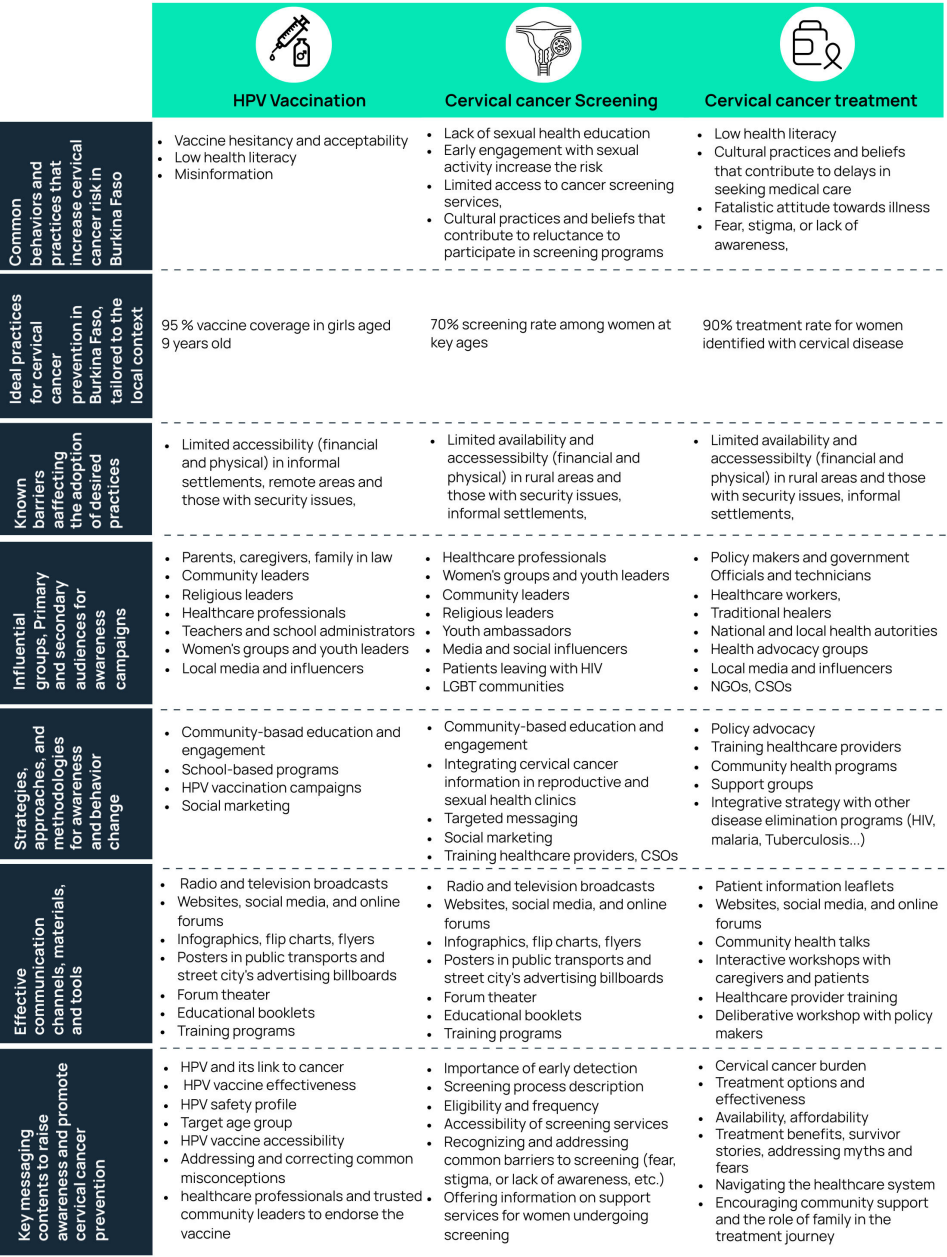
Summary of the discussions of a stakeholders workshop for the development of awareness messaging for cervical cancer prevention in Burkina Faso.

The five-day discussion resulted in a consensus strategy in line with multifaced steps that should be considered for raising awareness for cervical cancer elimination in Burkina Faso and reflects the global strategy. A summary of the discussion is presented in **Figure 2**.

## 4 Discussion

Unlike resource-rich settings where CC elimination strategies are well-established with access to vaccines, regular screening, and treatment for patients, the nascent CC elimination program in Burkina Faso is defined with limited infrastructure and resources, necessitating innovative, context-specific approaches. This workshop aimed to develop a consensus strategy to enhance public awareness of CC, leveraging a participatory approach. By gathering a diverse group of stakeholders, the event fostered a collaborative environment conducive to generating innovative, context-specific solutions for CC awareness and prevention (24).

Through the five-day discussions, we gained comprehensive insights and developed practical, stakeholder-driven solutions for cervical cancer awareness. These solutions are innovative and creative, adapted to the local context, and represent a significant step in advancing communication for cervical cancer elimination in Burkina Faso.

### 4.1 Addressing communication challenges with innovative solutions

The workshop highlighted several communication issues: 1) the lack of continuous mass media communication about cervical cancer prevention options available in Burkina Faso and their accessibility; 2) the lack of communication between caregivers (clinicians, oncologists, radiologists, nurses, psychologists, etc.) for optimal patient care; 3) the fact that communication with patients is not adapted to their needs and cultural habits and 4) the challenges related to communication on social networks, particularly in terms of authenticity and fact-checking. To address these challenges, an effective and low-cost strategy discussed during the workshop consists of inclusive and integrative communication and healthcare programs for cancer prevention. These programs should be designed to be innovative and practical, ensuring they can be easily integrated into everyday healthcare practices (25). Lessons learned from other disease areas, such as HIV/AIDS, can provide effective inspiration, working models to build capacity, and communication strategy to improve access, increase efficiency, and ultimately contribute to better health outcomes in vulnerable populations. The following practical approaches that can be easily integrated into everyday healthcare practices, drawing inspiration from successful HIV/AIDS strategies, were recommended:

- Comprehensive education campaigns that address both men and women across different life stages. It should leverage lessons from HIV/AIDS education by emphasizing the importance of regular screenings, and HPV vaccination and use testimonials from survivors and patients to personalize the message and increase its impact. These communications should emphasize the importance of CC early detection which improves cancer outcomes by enabling care at the earliest possible stage and potentially better outcomes for patients (26, 27).
- Active engagement of male partners in CC prevention programs similar to HIV/AIDS strategies, educating them on the importance of supporting their partners in getting screened and vaccinated, and on their role in preventing HPV transmission. Encourage men to get their daughters vaccinated against HPV and like strategies used in HIV prevention, encouraging them in condom use and regular testing.
- Integration of CC screening and HPV vaccinations into routine health services, such as family planning, prenatal visits, and HIV testing and counseling sessions to mirror the HIV model where testing is often integrated into other health services to reduce stigma and increase accessibility. Use mobile clinics and outreach to offer CC screening and HPV vaccinations in remote or underserved areas, drawing from the HIV/AIDS strategy of reaching out to populations with limited access to healthcare facilities.

A systematic review has shown that integration of cervical cancer screening and treatment with HIV services using different models of service delivery is feasible as well as acceptable to women living with HIV (28). An innovative project for health promotion by primary healthcare professionals was tested in primary healthcare centers of the Basque Healthcare Service. It focuses on promoting multiple healthy habits and demonstrates the feasibility of implementing health promotion programs in routine primary health care (29). In a scarce resource setting like Burkina Faso, inclusive and integrative healthcare programs may not generate extra costs for the health sector (30). However, it requires investment in nationally led and evidence-based capacity-building activities in participatory approaches to cancer policies for civil society organizations like cancer patients’ organizations (31, 32).

During the workshop, the participants agreed on community awareness strategies tailored to the country’s socio-cultural context using relevant and accessible content, age-appropriate language, and mediums such as:

- Mass communication primarily aims to reach a broad audience: social networks, billboards, theaters, fairs, flyers, posters, informational booklets, advice cards, models, pamphlets, interactive radio talks, television series, and films.
- Interpersonal communication that involves establishing a direct relationship between a health professional and a patient, or between two patients. This allows for providing emotional support, giving advice, explaining different treatments, and answering patient questions. The communication tools also include brochures, fact sheets, documents, flipcharts, roll-up banners, etc.
- Digital communication is very widespread and can reach a wide audience but it can also be personalized to meet individual needs. The communication tools for this strategy include social networks, websites, mobile apps, chatbots, etc.
- A community-based approach that requires community involvement in CC elimination activities. This can involve raising awareness, disseminating information among community members, recommending health centers or specialists, etc. The communication tools for this strategy include theater and sketches, community radio talks, brochures, and posters.

### 4.2 Meaningful engagement with patients

Meaningful engagement with cancer patients and their relatives was highlighted during the workshop to help them understand their disease, and treatment options through the different phases of their treatment for improved quality of life. This was reported to be important for the patients’ medical outcomes and also the overall well-being of their support systems (32). Indeed, the meaningful engagement of patients, survivors, caregivers, or families forms a vital part of the lived experience of those affected by cancer. It is essential to meet the information needs of cancer patients and their caregivers. It can reduce caregiver burden, improve physical and mental health, and promote intimacy. According to Samson et al. (2022) (30), cancer patients’ organizations should be recognized and considered as a critical voice in national cancer policies in LMICs as part of the right to health but also as a prerequisite to quality cancer policies.

### 4.3 Next steps

Follow-up meetings are planned to validate and monitor the dissemination of developed messages. The workshop aligns with WHO’s strategy for CC elimination and emphasizes the critical role of localized health initiatives.

It was also suggested to create a network of health professionals to facilitate communication among them, with the community and the patients. This can be facilitated with the following approaches:

- Digital tools that could be developed to allow more efficient collaboration: data-sharing platforms, and online discussion spaces, among others, would be of great benefit.
- Continuous public awareness campaigns on local radio, television, and social networks around the HPV vaccine, CC screening, and the importance of fact-checking could be carried out to combat the spread of fake news.
- Development and implementation of training programs for health professionals, teaching them to tailor their communication to the needs and cultural sensibility of their patients.

## 5 Conclusion

This stakeholder’s workshop aligns with WHO’s strategy for CC elimination (12) and emphasis on community-level interventions. It contributes to the global efforts in combating cervical cancer, emphasizing the critical role of localized health initiatives. Participants in the workshop came from various backgrounds and had varying levels of understanding about cervical cancer elimination strategy and different perspectives and opinions. This led to multiple challenges during the discussions. However, the group sessions provided each participant with an equal opportunity to voice their opinions. The four skilled moderators also played an important role in guiding the group discussions and finding common ground among differing viewpoints. Despite potential participant selection biases, the collaborative approach provided comprehensive insights for CC awareness strategies in Burkina Faso, offering valuable guidance for national health authorities. It represents a significant step towards CC elimination in Burkina Faso. The next steps should be the development and dissemination of the messages initiated during the workshop involving all stakeholders, an assessment of their impact over time, and an evaluation of their effectiveness for CC control. The collaborative efforts initiated during the workshop must be sustained and expanded upon to make significant strides in the strategy for CC elimination in the region.

## Data availability statement

The raw data supporting the conclusions of this article will be made available by the authors, without undue reservation.

## Author contributions

SO: Writing – review & editing, Writing – original draft, Visualization, Validation, Supervision, Resources, Project administration, Methodology, Investigation, Funding acquisition, Formal Analysis, Data curation, Conceptualization. AB: Writing – review & editing, Writing – original draft, Visualization, Validation, Investigation. GT: Writing – review & editing, Writing – original draft, Visualization, Validation, Resources, Methodology, Investigation, Data curation, Conceptualization. SK: Writing – review & editing, Writing – original draft, Visualization, Validation, Supervision, Project administration, Methodology, Formal Analysis, Conceptualization. AT-T: Writing – review & editing, Visualization, Validation, Methodology, Investigation. MM: Writing – review & editing, Visualization, Validation, Methodology, Investigation. SK: Writing – review & editing, Visualization, Supervision, Project administration, Investigation. BS: Writing – review & editing, Visualization, Validation, Supervision, Resources, Project administration, Methodology, Investigation, Funding acquisition, Conceptualization. MD: Writing – review & editing, Validation, Resources, Project administration, Methodology, Investigation, Funding acquisition, Data curation. OL: Writing – review & editing, Visualization, Validation, Supervision, Resources, Project administration, Methodology, Investigation, Funding acquisition, Conceptualization. NM: Writing – original draft, Visualization, Validation, Supervision, Resources, Project administration, Methodology, Investigation, Funding acquisition, Formal Analysis, Data curation, Conceptualization. JS: Writing – review & editing, Writing – original draft, Visualization, Validation, Supervision, Software, Resources, Project administration, Methodology, Formal Analysis, Data curation, Conceptualization.
