# Supplementary material for: Cervical cancer prevention in Burkina Faso: a stakeholder’s collaboration for the development of awareness messaging
Source: Front Oncol. 2024 May 10;14:1383133. doi: 10.3389/fonc.2024.1383133 (PMC11116777; doi:10.3389/fonc.2024.1383133)
Supplement: Supplementary file 1 [file DataSheet_1.docx]

**Supplementary Table 1: Behavioral Analysis template and Development of awareness messaging for cervical cancer prevention in Burkina Faso**

**Guidelines for the items to address:**

**What is the specific, feasible, and effective behavior to promote?**

**A behavior is:**

**An action**

**Observable**

**Specific (time, place, quantity, duration, frequency)**

**Measurable**

**Feasible**

**Behavior Characterization Table**

| **Easier to Adopt** | **More Difficult to Adopt** |
| --- | --- |
| Once | Continuous or frequent |
| No skills needed | Complex skills needed |
| Immediate positive results | Delayed outcome (or immediate negative result) |
| No cost | High cost |
| Little time | A lot of time |
| Fits social norms | Against social norms |
| Resources always available | Resources rarely available |
| Requires one person | Requires multiple people |
| Single step | Multiple steps |
| Low tech | High tech |

**Who are the Priority Groups and Influence Groups? (Describe in six different ways)**

- Six ways to describe target groups:
  1. Demographic characteristics
  2. Daily routine: How most people spend their days
  3. Something most group members want
  4. Something that prevents the group from practicing the Behavior (barriers)
  5. What the Priority Group knows, feels, and practices regarding the Behavior
  6. The willingness of most group members to adopt the new Behavior (Stage of Change)

**Definitions:**

- **Priority Group(s):** The Priority Group(s) will practice the positive Behavior (e.g., farmers, mothers of children under five, village youth). The Priority Group can be a community member (e.g., a mother, a farmer) or a service provider (e.g., an extension agent, micro-credit provider, community health worker).
- **Influence Group(s):** The Influence Group(s) influence the Priority Group(s) in the area of Behavior and can either support or hinder the Priority Group from adopting positive Behaviors. This group is typically identified through research among the Priority Group.

**What are the most important Determinants affecting this Behavior in this group?**

- Environmental factors, psychosocial factors, social norms, predisposing factors (Social and behavioral determinants include: self-efficacy, perceived social norms, perceived positive consequences, perceived negative consequences, access, cues for action/reminders, perceived susceptibility, perceived severity, perceived divine will, politics, and culture).

**What are the Bridges to Activities that need to be promoted?**

**What are the Activities that will be executed to address the Bridge to Activities?**

**Complete the following table:**

| **Common behaviors and practices that increase cervical cancer risk in Burkina Faso** | **Ideal practices for cervical cancer prevention in Burkina Faso, tailored to the local context** | **Known barriers affecting the adoption of desired practices** | **Influential groups, primary and secondary audiences for awareness campaigns** | **Strategies, approaches and methodologies for awareness and behavior change** | **Effective communication, channels, materials, and tools** | **Key messaging contents to raise awareness and promote cervical cancer prevention** |
| --- | --- | --- | --- | --- | --- | --- |
|  |  |  |  |  |  |  |
|  |  |  |  |  |  |  |
|  |  |  |  |  |  |  |
|  |  |  |  |  |  |  |
